# Multilocus Sequence Typing of Pathogenic *Candida albicans* Isolates Collected from a Teaching Hospital in Shanghai, China: A Molecular Epidemiology Study

**DOI:** 10.1371/journal.pone.0125245

**Published:** 2015-04-28

**Authors:** Kefei Wu, Tao Luo, Li Li, Qiangqiang Zhang, Junhao Zhu, Qian Gao, Min Chen, Min Zhu

**Affiliations:** 1 The Center for Medical Mycology, Department of Dermatology, Huashan Hospital, Fudan University, Shanghai, 200040, People’s Republic of China; 2 Key Laboratory of Medical Molecular Virology, Institutes of Biomedical Sciences and Institute of Medical Microbiology, Fudan University, Shanghai, 200032, People’s Republic of China; 3 Shanghai Key Laboratory of Molecular Medical Mycology, Department of Dermatology, Shanghai Changzheng Hospital, Second Military Medical University, Shanghai, 200003, People’s Republic of China; National Center for Biotechnology Information (NCBI), UNITED STATES

## Abstract

Molecular typing of *Candida albicans *is important for studying the population structure and epidemiology of this opportunistic yeast, such as population dynamics, nosocomial infections, multiple infections and microevolution. The genetic diversity of *C*. *albicans *has been rarely studied in China. In the present study, multilocus sequence typing (MLST) was used to characterize the genetic diversity and population structure of 62 *C*. *albicans *isolates collected from 40 patients from Huashan Hospital in Shanghai, China. A total of 50 diploid sequence types (DSTs) were identified in the 62 *C*. *albicans *isolates, with 41 newly identified DSTs. Based on cluster analysis, the 62 isolates were classified into nine existing clades and two new clades (namely clades New 1 and New 2). The majority of the isolates were clustered into three clades, clade 6 (37.5%), clade 1 (15.0%) and clade 17 (15.0%). Isolates of clade New 2 were specifically identified in East Asia. We identified three cases of potential nosocomial transmission based on association analysis between patients’ clinical data and the genotypes of corresponding isolates. Finally, by analyzing the genotypes of serial isolates we further demonstrated that the microevolution of *C*. *albicans* was due to loss of heterozygosity. Our study represents the first molecular typing of *C*. *albicans *in eastern China, and we confirmed that MLST is a useful tool for studying the epidemiology and evolution of *C*. *albicans*.

## Introduction

As an opportunistic fungal pathogen, *Candida albicans* often causes cutaneous or mucosal infections. It can lead to superficial infection as well as deep infections in the lungs, intestinal tract or even blood, particularly when the host is debilitated or immunocompromised [1, 2]. Molecular epidemiology that combines traditional epidemiological investigation with molecular typing is useful for identifying community or nosocomial infections and tracing the source of transmission and outbreaks. Several genotyping methods have been used to study the molecular epidemiology of *C*. *albicans*, including pulsed-field gel electrophoresis (PFGE), restriction fragment length polymorphism (RFLP), random amplified fragment length polymorphism (RAPD), and multilocus sequence typing (MLST) [3]. Assays based on PFGE, RFLP or RAPD, however, are labor-intensive and time-consuming. Furthermore, the results of these methods are difficult to compare among laboratories. By contrast, MLST, as a relatively new tool based on DNA sequencing, exhibits high discriminatory power and reproducibility, which overcome the flaws of more subjective methods, making it possible to compare results among laboratories [4].

Recently, a standard MLST protocol for molecular characterization of *C*. *albicans* has been proposed based on the sequences of seven housekeeping genes (*AAT1a*, *ACC1*, *ADP1*, *MPI1b*, *SYA1*, *VPS13* and *ZWF1b*) [5]. This method has been widely used to study the population structure, transmission and microevolution of *C*. *albicans*. Based on MLST, a total of 18 clades have been identified worldwide and these clades are associated with the geographic distribution of *C*. *albicans* [3, 6, 7]. A remarkable difference in genotypic patterns has been reported between the vaginal isolates of *C*. *albicans* from Chinese and non-Chinese women [8]. Only 11.3% of the non-Chinese vaginal isolates were grouped into the cluster concentrated with Chinese vulvovaginal candidiasis isolates. Isolates of different genotypes or clades may have different phenotypes in terms of drug resistance, virulence and pathogenesis. A large proportion of flucytosine-resistant isolates were grouped into clade 1, and the mechanism is the mutation of a single nucleotide in the *FUR1* gene [9]. While isolates from different anatomical sites have no significantly common characteristics [10, 11], some isolates are predominately identified from a specific group of patients. A study of the *C*. *albicans* isolates in the digestive tract of dyspeptic patients revealed a possible link between particular *C*. *albicans* genotypes and the host microenvironment [12]. For example, the *C*. *albicans* genotype *dst1593* was predominately identified from *C*. *albicans* isolates in the digestive tract of a group of patients with dyspepsia but not a healthy group. Furthermore, Bougnoux *et al*. provided evidence of microevolution of *C*. *albicans* within one person based on MLST [11].

So far, several epidemiological studies of *C*. *albicans* based on MLST have been conducted in China. A study in Beijing reported the epidemiological characteristics of *C*. *albicans* collected from women with genital candidiasis [8]. The genetic diversity of *C*. *albicans* causing bloodstream infections in women in the intensive care unit of West China Hospital, Sichuan University, was analyzed by MLST in 2012 [13]. Furthermore, a study of *C*. *albicans* sampled from the digestive tract of dyspeptic patients was conducted in Shenyang [12]. However, no epidemiological study of *C*. *albicans* has been carried out in Shanghai, eastern China. In this study, we used MLST to study the genetic diversity and population structure of 62 isolates from 40 patients in Shanghai, China. Furthermore, the MLST results were used to identify potential nosocomial infections and explore the evidence of microevolution.

## Materials and Methods

### Ethics statement

This study was approved by the institutional review board of Huashan Hospital, Fudan University (Grant No. KY2014-219). This study was an identity-unlinked survey on the remnants of clinical samples obtained from inpatients, so the need to obtain consent was waived. No participants were recruited or followed-up during the course of this study. All the data were analyzed anonymously.

### *C*. *albicans* isolates

A total of 62 *C*. *albicans* isolates from 40 patients were collected during September to November in 2012 in the Center for Medical Mycology of Huashan Hospital. All the isolates were cultured from sputum or urine samples and were positive of direct microscopic examination, indicating they are pathogenic *C*. *albicans*. In addition, all the isolates showed green colonies on CHROMagar Candida medium (CHROMagar). Colonies were collected for genomic DNA extraction after subculture on Sabouraud agar for 48 hours.

### DNA extraction

Two sweeps of colonies were put into 150 μL Tris-EDTA buffer, boiled for 15 minutes and then centrifuged at 13,000 rpm for 10 minutes. Supernatant that contained genomic DNA of *C*. *albicans* was used as template for polymerase chain reaction (PCR) assay.

### DNA amplification and sequencing

PCR assays were carried out in 25 μL reaction volumes containing 1 μL of extracted DNA, 1 μL of each primer (10 mM), 12.5 μL of 2× PCR Master Mix (TianGen) and 9.5 μL double-distilled water. PCRs were performed with an initial 5-min denaturation step at 95°C, followed by 35 cycles of 94°C for 40 s, 52°C for 40 s, and 72°C for 45 s, with a final extension step of 10 min at 72°C. The primers used in PCR assays were from the previous study for MLST analysis of *C*. *albicans* [5]. Bi-directional DNA sequencing reactions were completed by a DNA analyzer (ABI 3730XI).

### Allelic and cluster analyses

We analyzed the allelic status (homozygote or heterozygote) of each nucleotide based on the chromatograms using *Geneious* software, version 5.6.5 (Biomatters Ltd). For a diploid genome, the base at each polymorphic site can be homozygous or heterozygous. So the bases were doubled when the site was homozygous and written in pairs when heterozygous [7]. The DNA sequences of the seven housekeeping genes were concatenated for cluster analysis and clade definition as previously described [14]. A dendrogram was constructed based on the unweighted-pair group method using average linkages and the software package *MAGA*, version 6.0, as described previously [15].

## Results

Among the 62 isolates included in this study, 28 were isolated from different patients, and 34 were serially isolated from 12 patients. Five isolates (5/62, 8.1%) were cultured from urine samples and the remaining 57 isolates were collected from sputum samples. Of the 37 inpatients, 19 (19/37, 51.4%) were admitted for the primary diseases in lung. Among these 19 patients, 16 (16/19, 84.2%) were positive of *C*. *albicans* in sputum, and other 3 (3/19, 15.8%) were positive of *C*. *albicans* in urine. However, only one inpatient was hospitalized because of primary candidiasis. Most of patients were secondarily infected by *C*. *albicans*. (S1 Table) All 62 isolates were confirmed as *C*. *albicans* by sequencing the internal transcribed spacer region.

### Genetic diversity of *C*. *albicans* isolates based on MLST

DNA sequences of the coding regions of seven housekeeping genes were concatenated to generate a dataset of 2,883 bp for each isolate. A total of 61 variable loci were identified among the 62 isolates, representing 2.1% (61/2,883) of the total nucleotides. The concatenated sequences of 62 isolates were classified into 50 diploid sequence types (DSTs). We identified one, two and two new sequence types (STs) in loci *AAT1a*, *ACC1* and *VPS13*, respectively. One of the five new STs (*VPS13* in isolate 127188) was found to be a new combination of previously identified polymorphic loci, while the other four new STs represented new polymorphic loci. A G→K at position 300 in the *AAT1a* gene in isolate 129273 is relative to the reference start position. Isolates 126295 and 126408 were obtained from the same patient: 126295 had C→Y at position 78, and 126408 had C→T at the same position in *ACC1*. The nucleotide at this position in the *ACC1* gene of other isolates registered in the database were all C, so the T might result from mutation, while the isolate 126295 whose locus was Y (C/T) may be the result of mating between haploids (C and T). For *VPS13*, isolate 126407 had C→Y at position 246 and isolate 127188 had A→W at position 134, which were not novel polymorphisms but a combination of polymorphic loci previously identified. These new alleles have been submitted to the MLST database (http://calbicans.mlst.net/) and the sequences of all isolates in this study can be retrieved from the database (http://calbicans.mlst.net/sql/sthtml.asp#) according to the STs of the housekeeping genes (S2 Table).

**Fig 1:**
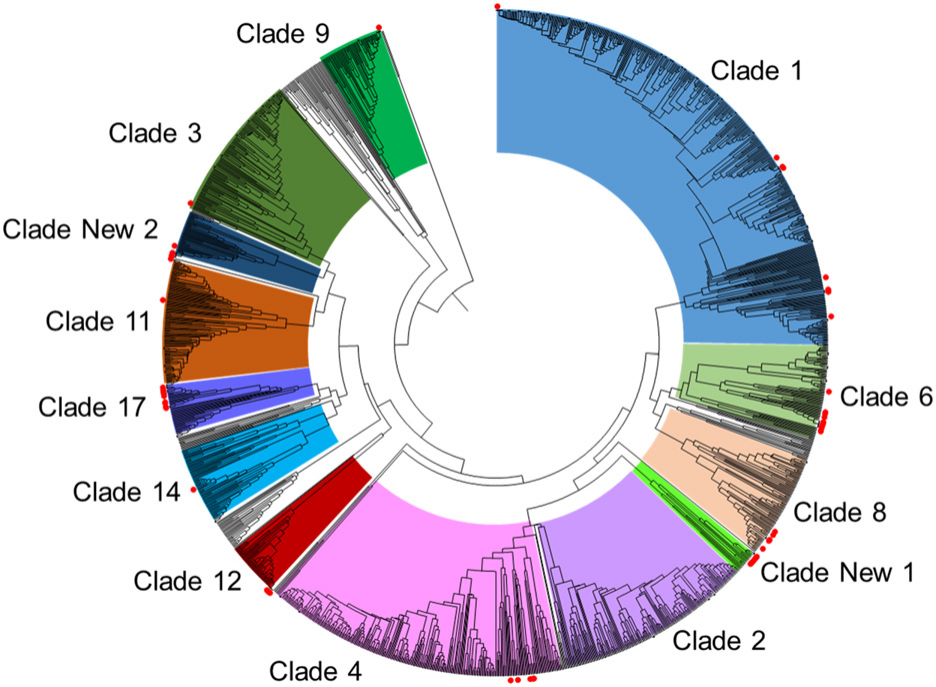
Genetic diversity of the 62 *Candida albicans* isolates. The phylogram was constructed from UPGMA analysis of uncorrected *P*-distances based on concatenated sequence of the seven loci. Sixty-two isolates in this study and a global collection of 1,563 isolates retrieved from the MLST database (http://calbicans.mlst.net) were included in the analysis. The major clades of global *C*. *albicans* are indicated. The isolates from this study were labeled as red dots to show their phylogenetic distribution.

**Fig 2:**
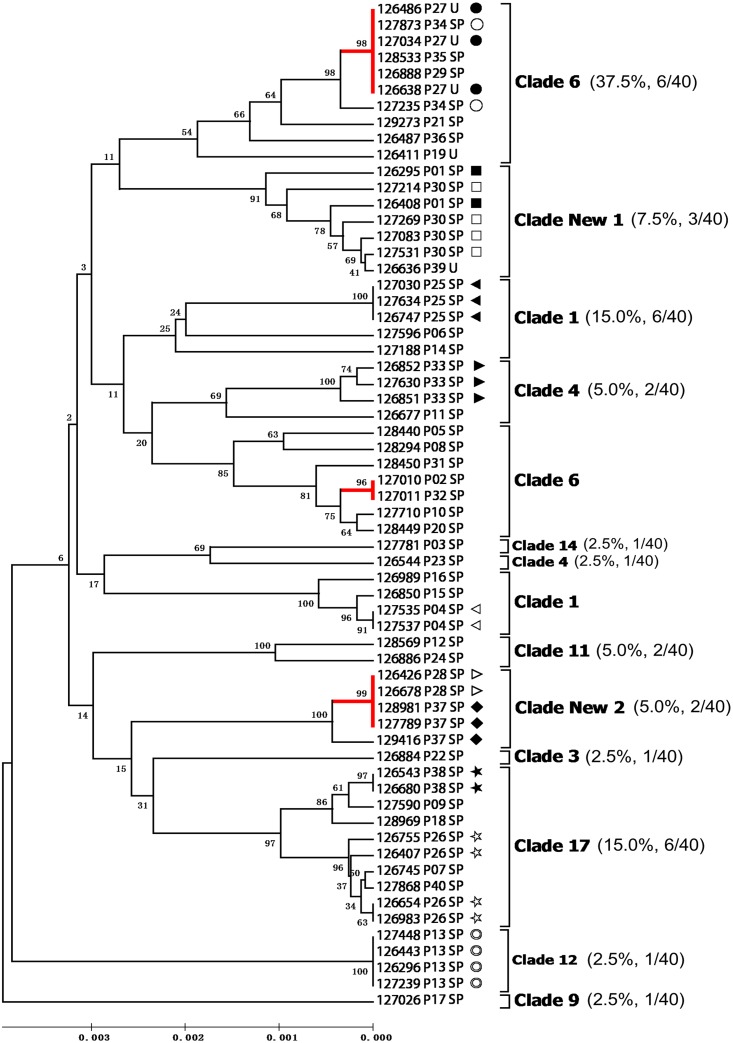
Potential nosocomial infections and microevolution events among the 62 *Candida albicans* isolates. The phylogram was constructed from UPGMA analysis of uncorrected *P*-distances based on concatenated sequence of the seven loci. Bootstrap values were calculated based on 1,000 duplications. Branches with identical DSTs are labeled in red. Serial isolates from the same patients were labeled with specific symbols. Clade assignments and their relative prevalence were indicated in the right column.

In order to group the 62 isolates into clades, we performed a cluster analysis of the 62 isolates included in the present study and 1,563 isolates deposited in the MLST database (http://calbicans.mlst.net). The 1,563 isolates in the MLST database were grouped into 17 clades (clusters with more than 10 isolates) or as singletons (clusters with fewer than 10 isolates), as previously identified. Of the 62 isolates, 50 isolates were clustered into 10 known clades (Fig 1). The remaining 12 isolates were grouped into two clusters with other isolates previously assigned as singletons. Given that both clusters contained over 10 isolates, they were named as clades New 1 and New 2. Isolates in clade New 1 was rare in the database (12/1,563, 0.8%). Only seven isolates from three patients in the present study were grouped into the clade New1. Clade New 2 mainly contained isolates that were previously collected from Korea [15]. Four isolates from two patients in the present study were clustered with these Korean isolates in clade New 2, suggesting that this new clade may be specific in East Asia. In order to evaluate the prevalence of each clade in the hospital, we took only one isolate for each patient (the initial isolate) for analysis. The most prevalent clades in our hospital were clade 6 (15/40, 37.5%), followed by clades 17 (6/40, 15.0%) and 1 (6/40, 15.0%). The frequencies of other clades were relatively low (Fig 2).

### Nosocomial infections

It is worth noting that some patients shared the same DST, which might be evidence for nosocomial transmission when the data are analyzed with the epidemiological findings [16]. Three DSTs, each of which was shared by isolates from more than one patient, were identified (Fig 2). Isolates from patients P28 and P37 shared a new DST of clade New 2. Although these two patients were in different wards, the period of their stays in the hospital overlapped, suggesting potential transmission. Patients P02 and P32 shared a DST of clade 6. However, a number of isolates from these two patients were serially isolated, which may result from either contamination during culturing or transmission. Isolates from patients P27, P29 and P34 shared a DST of clade 6. These three patients were in the hospital at the same period of time. It is interesting that patients P29 and P34 stayed in the same wards at the same time, which is highly suggestive of transmission between them. As to patient P35, who was an outpatient and had no epidemiological link with other patients in this study, he may have become infected in the community.

### Microevolution at the MLST loci

To identify potential microevolution of *C*. *albicans* within patients, we compared the DSTs of multiple isolates that were isolated from the same patients. Serial isolates were collected from 12 patients within 1 to 24 days. For six patients (6/12, 50.0%), the isolates from each of them were identical, while differences were detected in the remaining six patients (Fig 2). A total of 30 loci were detected in the seven genes (Table 1). Surprisingly, all variations between isolates from the same patients resulted from loss of heterozygosity (LOH). For patients P26, P33 and P37, the variations could be explained by LOH between the collected isolates. For the other three patients P01, P30 and P34, the variations were most likely caused by LOH between the collected isolates and other intermediate colonies that were not isolated in this study.

**Table 1:** Polymorphisms on the seven MLST markers in the six patients with repeated sampling and the isolates that failed to share the same DSTs.

Patient no.	Isolate no.	*AAT1a*			*ACC1*						*ADP1*	*MPI1b*											*SYA1*				*VPS13*				*ZWF1b*
		40	70	124	78	179	211	281	317	392	35	27	34	36	66	72	94	107	234	237	276	289	1	25	61	351	217	241	282	370	482
P01	126295				C/T	C/T		C/T									G	C	A	G	A	G					T	G	A	T	
	126408				T	T		T									A/G	A/C	A/C	A/G	A/G	A/G					C/T	A/G	A/G	A/T	
P26	126407						A/C		C/T															A	G						
	126654/126983						A/C		C/T															A/C	A/G						
	126755						A		C															A/C	A/G						
P30	127083					C/T		C/T	C			G	A	C	G	T	A/G	A/C													A/T
	127214					C		C	C/T			A/G	A/G	C/T	G/T	A/T	G	C													A
	127269					C		C	C/T			G	A	C	G	T	A/G	A/C													A
	127531					C/T		C/T	C			G	A	C	G	T	A/G	A/C													A
P33	126851									C/T	A/G												C/T	A/C		C/T					
	126852									C/T	A												T	A		C					
	127630									C	A/G												T	A		C					
P34	127235	A/G	T	C/T																	G										
	127873	A	C/T	C																	A/G										
P37	127789/128981																		A/C	A/G	A/G	A/G									
	129416																		C	A	G	A									

## Discussion

We used MLST to characterize the genetic diversity and population structure of *C*. *albicans* isolated from a teaching hospital in Shanghai, China. All isolates were cultured from samples that were positive of direct microscopic examination, and they tended to be isolates causing infections rather than commensal isolates. In previous studies, clade 1 was recognized as the most prevalent clade globally, followed by clade 2 [3, 7]. In this study, clade 1 was found in a relatively large proportion of infected samples, and we did not obtain isolates belonging to clade 2; a finding that was similar to the results of a study in Brazil [6]. It is worth noting that clade 17, which was found in a relatively high proportion of isolates in this study (6/40, 15.0%), was also recognized as a highly frequent clade in a study including 44 bloodstream isolates in Hong Kong in 2000 (9/40, 22.5%). In the same year, the isolates belonging to clade 17 were mostly isolated in East Asian regions such as Taiwan and Japan [14]. It could be speculated that clade 17, showing lower isolation rates in other areas, might be more virulent or infectious for East Asian people. Twelve isolates in this study grouped into two clusters, with other isolates previously assigned as singletons, and we named them as clades New 1 and New 2. Like clade 17, isolates belonging to clade New 2 were clustered with isolates collected from Korea [15], suggesting that this new clade may be specific in East Asian areas. However, clade 6, which contained the largest proportion of isolates (15/40, 37.5%), has been relatively rarely isolated in other studies, including studies in several East Asian areas [14, 15], suggesting that it is a clade which is endemic specifically in Shanghai, China.

As a standard genotyping method, MLST is widely used in research in nosocomial infections and infection among family members. Using MLST, Cliff *et al*. [17] found that isolates obtained from patients could share the same DSTs with isolates obtained from staff working in the same ward, suggesting nosocomial infections. Bougnoux *et al*. analyzed *C*. *albicans* obtained from the digestive tract of people with or without family members with Crohn disease, and their results suggested that the infection was present among family members [11]. Most patients included in our study were inpatients, among whom some were under long-term hospitalization. Isolates collected from patients P28 and P37 shared the same DST, and isolates collected from patients P27, P29, P34 and P35 shared the same DST. It is worth noting that in the case of isolates from patients P29 and P34, which shared the same DST, the two patients stayed in the same ward at the same time, suggesting the possibility of transmission in the wards. Furthermore, although the identical isolates were obtained from different wards, the period of hospital stay of those patients overlapped, except for patient P35, who was an outpatient and likely became infected outside the hospital. As Asticcioli *et al*. have shown, shared use of medical apparatus and instruments could be a cause of nosocomial transmission between different wards [18]. It was indicated that there might be nosocomial infections in hospitals not only from direct contact between patients but also due to cross-infection from medical staff, apparatus, instruments, and so on.

We also noted evidence of microevolution. Previously, LOH was detected as differences in the concatenated sequences of serial isolates, which could be explained by point mutation or mitotic recombination events [19, 20]. In this study, the LOH events were all associated with more than four bases of difference. In some cases, a heterozygous status at all variant sites was found in one isolate and a homozygous status was found in the other (e.g. 127788/128981 and 129416 from patient P37). In such situation, the homozygous status could be explained by LOH between theses isolates and was mostly likely caused by mitotic recombination. In other cases (patients P01, P30, and P34), variations between isolates could not be explained by LOH between the isolates, although we also noticed a slight trend of LOH. They might have resulted from LOH between collected isolates and other intermediate colonies which were not collected in this study. For instance, for isolates from patient P34, there might exist an isolate in which all five variant sites are heterozygous and some of them microevolved to be homozygous via LOH. Bougnoux *et al*. have shown the existence of microevolution in a single person based on MLST [11]. Although point mutations might account for cases where only one polymorphic site has evolved, our results strongly suggest mitotic recombination events in the other cases, because the LOH events encompassed several polymorphic sites within the locus. Furthermore, haplotype analysis and isolation of intermediate colonies are needed to confirm microevolution in clinical isolates of *C*. *albicans*.

## Conclusions

In conclusion, we analyzed the molecular epidemiology of *C*. *albicans* isolates in a teaching hospital in Shanghai, China using MLST. The most common clade was clade 6, followed by clades 1 and 17. We also provided evidence of nosocomial infections and microevolution. Further studies might focus on the confirmation of microevolution, the mechanism of microevolution, and whether such microevolution would improve the fitness of *C*. *albicans*. It seems that clades 17 and New 2 may be more virulent and infectious in East Asian areas. Therefore, the phenotypic and genotypic differences between clades 17 and New 2 from East Asian and other ethnic groups could be compared and analyzed *in vivo* and *in vitro*. At the same time, more samples should be collected in Shanghai to investigate whether clade 6 is the most endemic clade in Shanghai or is just a chance finding in this study.

## Supporting Information

S1 Table The clinical and epidemiological data of 62 isolates from 40 patients. (DOC)

S2 Table The sequence type (ST) assignments of all 62 isolates included in this study. (DOCX)
