# Supplementary material for: Multilocus Sequence Typing of Pathogenic Candida albicans Isolates Collected from a Teaching Hospital in Shanghai, China: A Molecular Epidemiology Study
Source: PLoS One. 2015 Apr 28;10(4):e0125245. doi: 10.1371/journal.pone.0125245 (PMC4412568; doi:10.1371/journal.pone.0125245)
Supplement: S1 Table — (DOC) [file pone.0125245.s001.doc]

**S1 Table. The clinical and epidemiological data of 62 isolates from 40 patients.**

| **Patient no.** | **Isolate no.** | **Gender**  **(Female/Male)** | **Age (yr)** | **Diagnosis** | **Admission time (in 2012)** | **Ward - bed no.** | **Sampling time**  **(date in 2012)** | **Sampling site** | **Clade** |
| --- | --- | --- | --- | --- | --- | --- | --- | --- | --- |
| **P01** | 126295/126408 | M | 89 | COPD | 9.4~10.22 | 27-301 | 9.7/9.8 | Sputum | New 1 |
| **P02** | 127010 | M | 49 | PTE | 9.3~9.21 | 21-9 | 9.17 | Sputum | 6 |
| **P03** | 127781 | M | 88 | UTI | 9.19~10.9 | 30-501 | 9.25 | Sputum | 14 |
| **P04** | 127535/127537 | F | 83 | Bronchiectasis | 9.20~10.10 | 2-101 | 9.21 | Sputum | 1 |
| **P05** | 128440 | F | 29 | Pneumonia | 10.2~10.17 | 5-3 | 10.9 | Sputum | 6 |
| **P06** | 127596 | F | 59 | ABPA | 9.20~10.19 | 53-15 | 9.21 | Sputum | 1 |
| **P07** | 126745 | M | 60 | Cerebral trauma | 9.7~9.21 | 55-2 | 9.13 | Sputum | 17 |
| **P08** | 128294 | F | 76 | Cerebral trauma | 10.3~10.9 | 55-20 | 10.6 | Sputum | 6 |
| **P09** | 127590 | F | 85 | Pneumonia | 9.21~10.10 | 40-15 | 9.12 | Sputum | 17 |
| **P10** | 127710 | M | 40 | Eosinophilia | 9.19~10.16 | 51-7 | 9.24 | Sputum | 6 |
| **P11** | 126677 | F | 48 | SS | 9.10~9.14 | 10-34 | 9.12 | Sputum | 4 |
| **P12** | 128569 | M | 53 | - | - | - | 10.10 | Sputum | 11 |
| **P13** | 126296/126443/127239/127448 | M | 90 | COPD | Long-stay | 27-23 | 9.7 | Sputum | 12 |
| **P14** | 127188 | M | 59 | Cerebral trauma | 9.17~9.28 | 55-33 | 9.18 | Sputum | 1 |
| **P15** | 126850 | F | 30 | Meningeoma | 8.15~9.18 | 3-601 | 9.13 | Sputum | 1 |
| **P16** | 126989 | M | 70 | Colon cancer | 9.7~10.9 | 29-17 | 9.18 | Sputum | 1 |
| **P17** | 127026 | M | 86 | CCI | 9.6~10.8 | 29-7 | 9.17 | Sputum | 9 |
| **P18** | 128969 | F | 91 | Pneumonia | Long-stay | 19-26 | 10.15 | Sputum | 17 |
| **P19** | 126411 | M | 95 | Chronic bronchitis | 9.4~9.24 | 19-14 | 9.8 | Urine | 6 |
| **P20** | 128449 | M | 88 | Cervical vertebrae fracture | 10.6~10.26 | 30-10 | 10.9 | Sputum | 6 |
| **P21** | 129273 | F | 30 | Gastric cancer | 10.8~10.17 | 10-31 | 10.17 | Sputum | 6 |
| **P22** | 126884 | M | 54 | Hydrocephalus | 8.7~9.18 | 40-13 | 9.14 | Sputum | 3 |
| **P23** | 126544 | F | 59 | Bronchiectasis | 9.10~9.17 | 5-1 | 9.11 | Sputum | 4 |
| **P24** | 126886 | M | 86 | Chronic bronchitis | - | - | 9.14 | Sputum | 11 |
| **P25** | 127634/126747/127030 | N | 90 | Colon cancer | 8.16~10.4 | 29-25 | 9.13/9.24/9.24 | Sputum | 1 |
| **P26** | 126407/126654/126755/126983 | M | 76 | Coronary atherosclerotic heart disease | Long-stay | 30-7 | 9.8/9.11/9.11/9.13 | Sputum | 17 |
| **P27** | 126486/127034/126638 | M | 81 | Respiratory failure | 8.22~10.9 | 40-5 | 9.10/9.10/9.17 | Urine | 6 |
| **P28** | 126426/126678 | M | 52 | Pulmonary candidiasis | 9.4~9.27 | 51-2 | 9.10 | Sputum | New 2 |
| **P29** | 126888 | F | 82 | Chronic bronchitis | 9.7~9.28 | 27-21 | 9.14 | Sputum | 6 |
| **P30** | 127083/127214/127269/127531 | M | 62 | Pneumonia | 9.12~9.25 | 55-12 | 9.17/9.18/9.19/9.21 | Sputum | New 1 |
| **P31** | 128450 | F | 83 | CCI | 9.13~10.8 | 27-8 | 10.9 | Sputum | 6 |
| **P32** | 127011 | F | 66 | Bronchiectasis | 9.3~9.19 | 53-17 | 9.17 | Sputum | 6 |
| **P33** | 126851/126852/127630 | F | 65 | Pneumonia | 9.21~10.22 | 2-10 | 9.13/9.13/9.24 | Sputum | 4 |
| **P34** | 127235/127873 | M | 92 | COPD | Long-stay | 27-14 | 9.19/9.26 | Sputum | 6 |
| **P35** | 128533 | - | - | - | - | - | 10.9 | Sputum | 6 |
| **P36** | 126487 | M | 28 | CNSI | 9.6~9.24 | 40-17 | 9.10 | Sputum | 6 |
| **P37** | 127789/128981/129416 | M | 82 | IPT | Long-stay | 27-5 | 9.25/9.25/10.18 | Sputum | New 2 |
| **P38** | 126543/126680 | M | 89 | COPD | Long-stay | 19-22 | 9.11/9.12 | Sputum | 17 |
| **P39** | 126636 | F | 85 | Pneumonia | 9.5~9.19 | 40-19 | 9.11 | Urine | New 1 |
| **P40** | 127868 | M | 77 | - | - | - | 9.26 | Sputum | 17 |

* COPD: chronic obstructive pulmonary disease; PTE: pulmonary thromboembolism; ABPA: allergic bronchopulmonary aspergillosis; SS: Sjgren's syndrome; CCI: cerebral circulation insufficiency; CNSI: central nervous system infection; IPT: idiopathic thrombocytopenic purpura
