# Supplementary material for: Multilocus Sequence Typing of Pathogenic Candida albicans Isolates Collected from a Teaching Hospital in Shanghai, China: A Molecular Epidemiology Study
Source: PLoS One. 2015 Apr 28;10(4):e0125245. doi: 10.1371/journal.pone.0125245 (PMC4412568; doi:10.1371/journal.pone.0125245)
Supplement: S2 Table — (DOCX) [file pone.0125245.s002.docx]

**S2 Table. The ST assignments at each of seven MLST loci for all 62 isolates in this study.**

| **Isolate no.** | **Patient no.** | ***AAT1a*** | ***ACC1*** | ***ADP1*** | ***MPI1b*** | ***SYA1*** | ***VPS13*** | ***ZWF1b*** |
| --- | --- | --- | --- | --- | --- | --- | --- | --- |
| **126295** | P01 | 13 | **96**^*^ | 6 | 19 | 7 | 55 | 12 |
| **126408** | P01 | 13 | **97**^*^ | 6 | 108 | 7 | 105 | 12 |
| **127010/127011** | P02/P32 | 55 | 14 | 4 | 3 | 24 | 45 | 15 |
| **127781** | P03 | 6 | 26 | 21 | 50 | 53 | 109 | 13 |
| **127535/127537** | P04 | 2 | 5 | 5 | 4 | 2 | 6 | 5 |
| **128440** | P05 | 102 | 29 | 4 | 28 | 6 | 109 | 12 |
| **127596** | P06 | 23 | 26 | 5 | 3 | 57 | 3 | 110 |
| **126745** | P07 | 59 | 5 | 21 | 2 | 80 | 108 | 15 |
| **128294** | P08 | 77 | 5 | 6 | 2 | 43 | 112 | 12 |
| **127590** | P09 | 59 | 26 | 75 | 2 | 81 | 108 | 90 |
| **127710** | P10 | 55 | 26 | 4 | 14 | 6 | 45 | 15 |
| **126677** | P11 | 1 | 3 | 4 | 3 | 7 | 73 | 112 |
| **128569** | P12 | 60 | 10 | 21 | 1 | 7 | 11 | 15 |
| **126296/126443/127239/127448** | P13 | 21 | 17 | 21 | 19 | 27 | 83 | 22 |
| **127188** | P14 | 4 | 4 | 54 | 9 | 89 | **252**^*^ | 105 |
| **126850** | P15 | 2 | 2 | 5 | 4 | 2 | 6 | 5 |
| **126989** | P16 | 2 | 5 | 5 | 2 | 2 | 6 | 5 |
| **127026** | P17 | 62 | 3 | 3 | 3 | 3 | 39 | 95 |
| **128969** | P18 | 59 | 7 | 21 | 41 | 81 | 108 | 90 |
| **126411** | P19 | 4 | 4 | 6 | 2 | 96 | 32 | 15 |
| **128449** | P20 | 55 | 3 | 4 | 3 | 6 | 45 | 15 |
| **129273** | P21 | **147**^*^ | 26 | 48 | 41 | 34 | 32 | 15 |
| **126884** | P22 | 59 | 34 | 15 | 4 | 60 | 20 | 70 |
| **126544** | P23 | 24 | 3 | 6 | 4 | 30 | 45 | 1 |
| **126886** | P24 | 60 | 13 | 10 | 100 | 7 | 11 | 15 |
| **127634** | P25 | 13 | 26 | 5 | 3 | 73 | 20 | 12 |
| **126747/127030** | P25/P25 | 13 | 26 | 5 | 3 | 93 | 20 | 12 |
| **126407** | P26 | 59 | 5 | 21 | 2 | 2 | **253**^*^ | 15 |
| **126755** | P26 | 59 | 3 | 21 | 2 | 80 | **253**^*^ | 15 |
| **126654/126983** | P26/P26 | 59 | 5 | 21 | 2 | 80 | **253**^*^ | 15 |
| **127034** | P27 | 21 | 26 | 48 | 18 | 8 | 111 | 15 |
| **126486/126638/126888** | P27/P27/P29 | 21 | 26 | 48 | 18 | 31 | 111 | 15 |
| **126426/126678/127789/128981** | P28/P28/P37/P37 | 5 | 32 | 21 | 34 | 7 | 74 | 5 |
| **127083** | P30 | 13 | 80 | 6 | 108 | 7 | 105 | 100 |
| **127214** | P30 | 13 | 26 | 6 | 34 | 7 | 105 | 12 |
| **127269** | P30 | 13 | 26 | 6 | 108 | 7 | 105 | 12 |
| **127531** | P30 | 13 | 80 | 6 | 108 | 7 | 105 | 12 |
| **128450** | P31 | 55 | 14 | 4 | 2 | 6 | 45 | 15 |
| **126851** | P33 | 25 | 32 | 5 | 2 | 89 | 202 | 8 |
| **126852** | P33 | 25 | 32 | 6 | 2 | 156 | 202 | 8 |
| **127630** | P33 | 25 | 31 | 5 | 2 | 156 | 202 | 8 |
| **127873** | P34 | 21 | 26 | 48 | 18 | 31 | 111 | 15 |
| **127235** | P34 | 80 | 26 | 48 | 41 | 31 | 111 | 15 |
| **128533** | P35 | 21 | 26 | 48 | 18 | 31 | 111 | 15 |
| **126487** | P36 | 21 | 8 | 48 | 19 | 31 | 32 | 15 |
| **129416** | P37 | 5 | 32 | 21 | 2 | 7 | 74 | 5 |
| **126543** | P38 | 59 | 14 | 21 | 2 | 81 | 108 | 90 |
| **126680** | P38 | 59 | 29 | 21 | 2 | 81 | 108 | 90 |
| **126636** | P39 | 13 | 80 | 6 | 108 | 56 | 105 | 12 |
| **127868** | P40 | 137 | 5 | 21 | 2 | 80 | 108 | 15 |

* ST numbers in bold represent new STs identified in the study.
